# Supplementary material for: Peripheral and lung resident memory T cell responses against SARS-CoV-2
Source: Nat Commun. 2021 May 21;12:3010. doi: 10.1038/s41467-021-23333-3 (PMC8140108; doi:10.1038/s41467-021-23333-3)
Supplement: Supplementary file 1 — Supplementary Information [file 41467_2021_23333_MOESM1_ESM.pdf]

Supplementary Information

Peripheral and lung resident memory T cell responses against SARS-CoV-2.

Grau-Expósito et al.

Supplementary Fig. 1

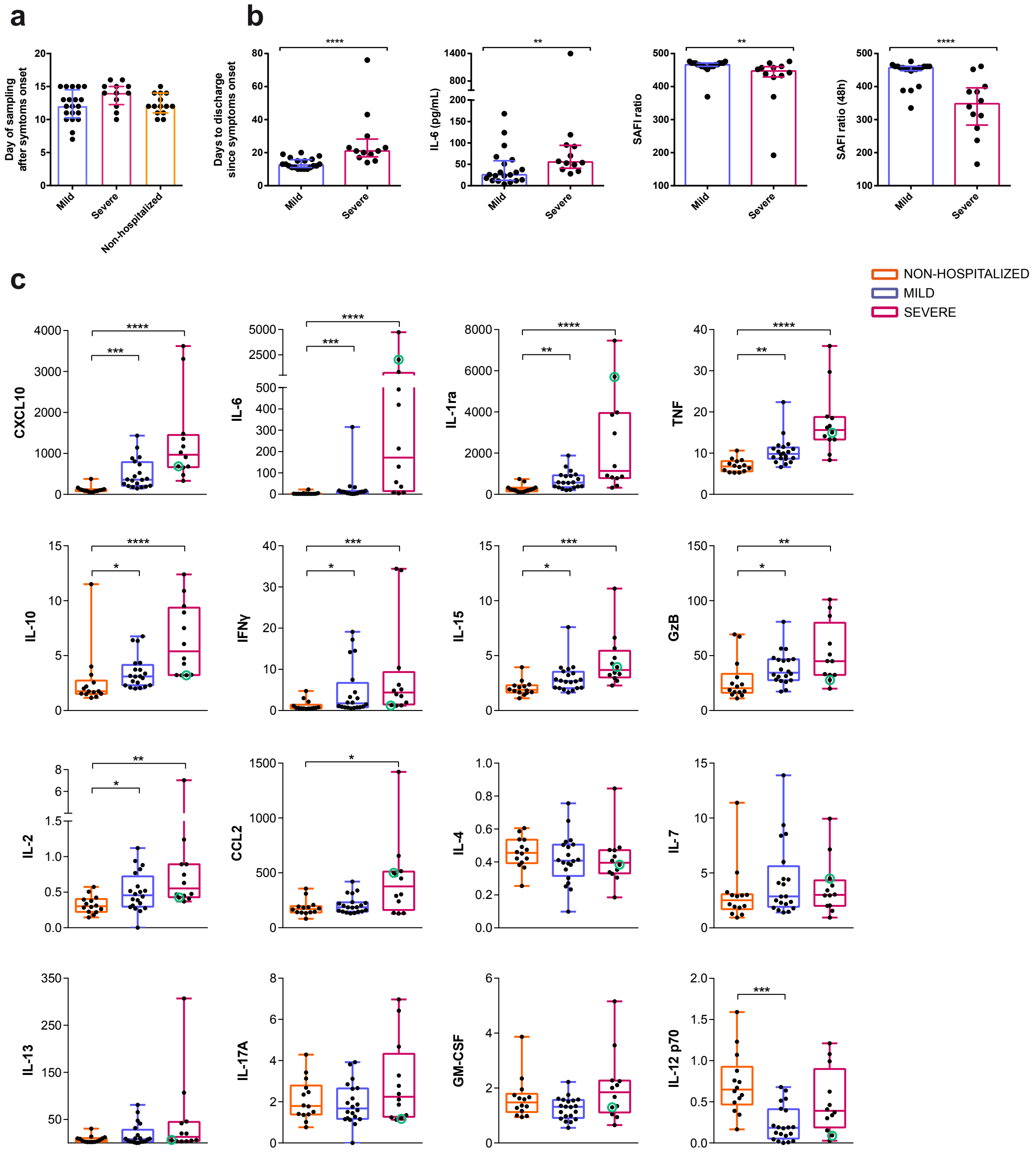

Supplementary Fig. 1. Clinical parameters and plasma cytokine levels during acute SARS-CoV-2 infection. (a) Day of sample analyses by study groups (non-hospitalized n=14 in orange; mild n=20 in blue and severe n=12 in pink). Boxes and error bars represent median and interquartile range (IQR). **(b)** Pairwise comparison between mild (n=20 in blue) and severe (n=12 in pink) hospitalized patients for several clinical parameters: days to hospital discharge since symptoms onset, baseline IL-6 levels (pg/mL) and SAFI ratio, which corresponds to the percentage of oxyhemoglobin saturation (SaO_2) in relation to the percentage of inspired oxygen (FiO_2), at baseline and after 48h. Boxes and error bars represent median and IQR. Statistical comparisons were performed using two-sided Mann-Whitney U tests (p<0.0001, p=0.008, p=0.005 and p=0.0002). **(c)** Plasma levels of chemokine (C-X-C motif) ligand 10 (CXCL10), interleukin (IL)-6, IL-1ra, tumor necrosis factor (TNF), IL-10, interferon (IFN)γ, IL-15, granzyme B (GzB), IL-2, C–C chemokine ligand 2 (CCL2), IL-4, IL-7, IL-13, IL-17A, granulocyte-macrophage colony stimulating factor (GM-CSF) and IL-12p70 in non-hospitalized (n=14) mild (n=20) and severe (n=12) COVID-19 patients. Green circle in the severe group indicates the deceased patient. Data are shown as individual patients and boxes and error bars represent median and IQR. Statistical comparisons were performed using Kruskal-Wallis rank-sum test with Dunn's multiple comparison test (two-sided): CXCL10 (p=0.0003 and p<0.0001), IL-6 (p=0.008 and p<0.0001), IL-1ra (p=0.004 and p<0.0001), TNF (p=0.003 and p<0.0001), IL-10 (p=0.044 and p<0.0001), IFNγ (p=0.021 and p=0.0003), IL-15 (p=0.037 and p<0.0001), GzB (p=0.028 and p=0.0032), IL-2 (p=0.049 and p=0.0009), CCL2 (p=0.030) and IL-12p70 (p=0.0003).

Supplementary Fig. 2

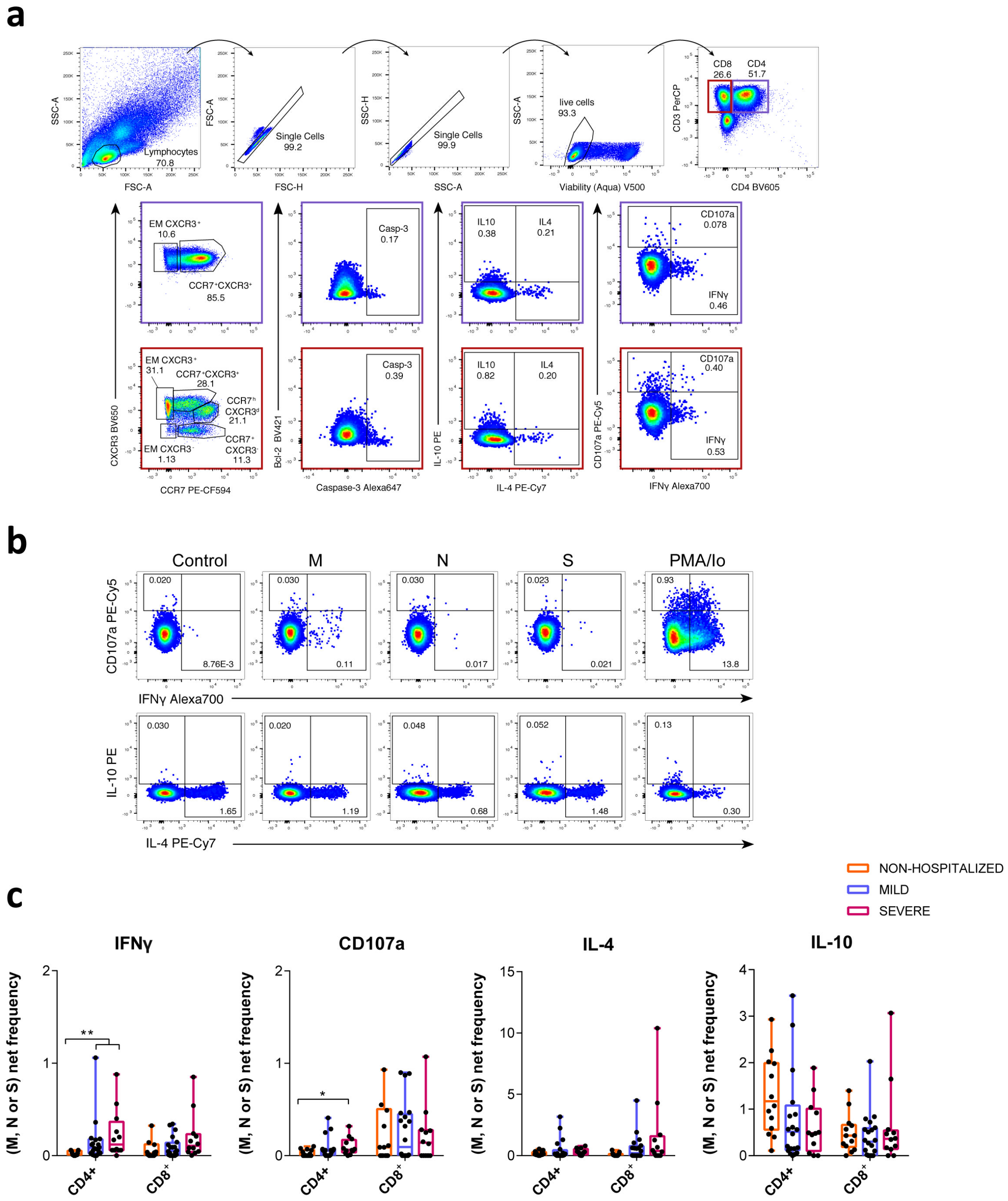

Supplementary Fig. 2. Gating strategy for the analyses of peripheral SARS-CoV-2-specific $CD4^+$ and $CD8^+$ T cells and overall T cell responses by clinical group. (a) General gating strategy for functional analysis of SARS-CoV-2-specific $CD4^+$ and $CD8^+$T cell populations presented in Figs. 1, 2, 3, 4 and 5. Gating strategy consisted of a lymphocyte gate based on FSC vs. SSC, followed by doublet and dead exclusion, live $CD3^+$T cell gate from where $CD4^+$ (purple) and $CD8^+$T cells (red) were gated. Out of these two T cell subsets (below), representative subsets based on their expression of CCR7 and CXCR3, the expression of caspase-3 and the expression of diverse functions among $CD4^+$ (top) and $CD8^+$T cells (bottom) are shown. **(b)** Representative flow cytometry plots showing individual functions after stimulation with the negative control, viral proteins (membrane (M), nucleocapsid (N) and spike (S)) and PMA/Ionomycin are shown for $CD4^+$T cells from a severe patient. **(c)** Comparison of the net frequency (background subtracted) of interferon (IFN)γ, CD107a, interleukin (IL)-4 and IL-10 expression in SARS-CoV-2-specific $CD4^+$ and $CD8^+$T cells in response to all viral proteins (membrane (M), nucleocapsid (N) and spike (S)) between study groups (non-hospitalized n=14 in orange; mild n=20 in blue and severe n=12 in pink). Data are shown as individual patients and boxes and error bars represent median and interquartile range (IQR). Statistical comparisons were performed using Kruskal-Wallis rank-sum test with Dunn's multiple comparison test (two-sided): IFNγ ($p=0.009$ and $p=0.0016$) and CD107a ($p=0.048$).

Supplementary Fig. 3

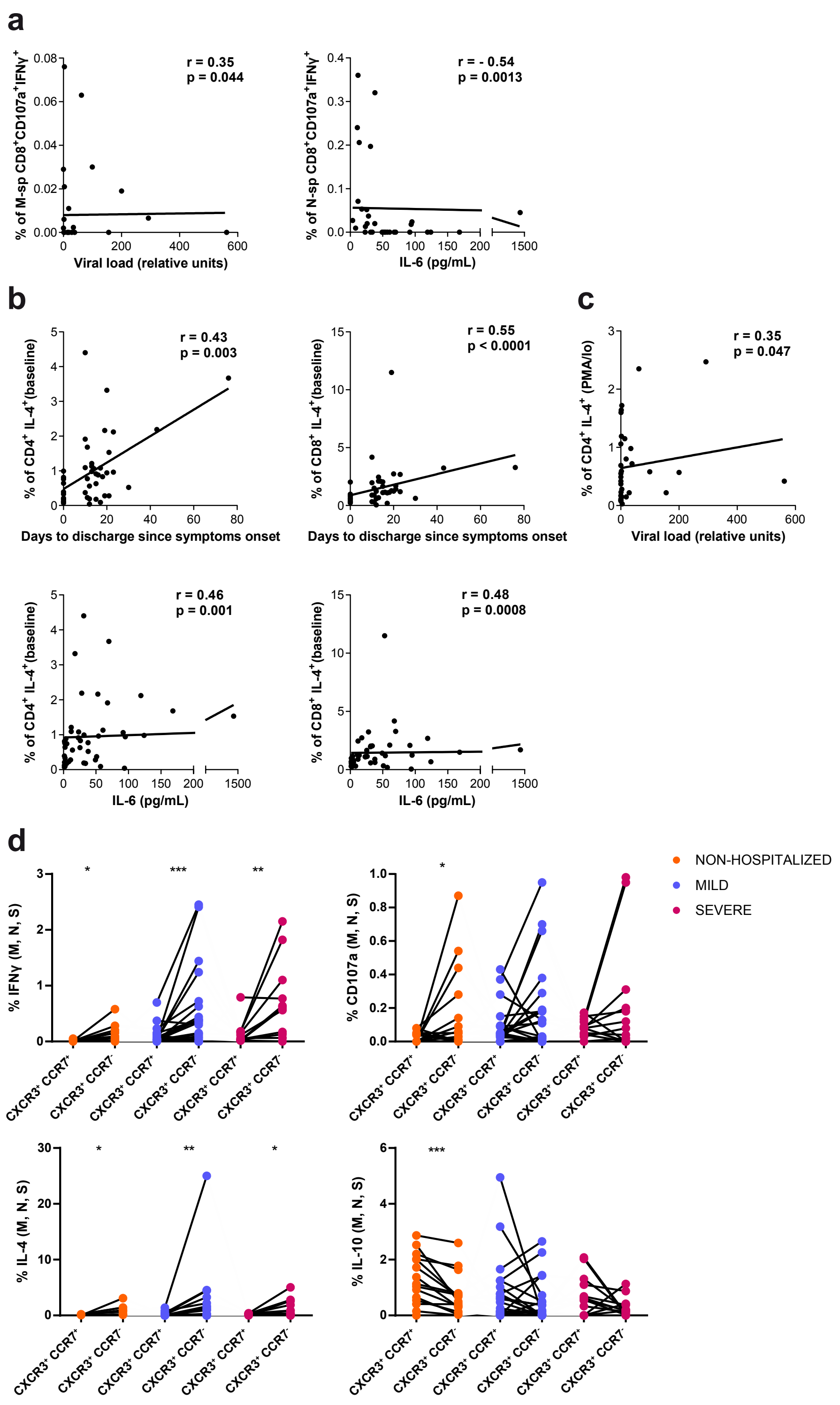

Supplementary Fig. 3. Correlations between peripheral SARS-CoV-2-specific T cells and clinical parameters, and migratory patterns of overall SARS-CoV-2-specific $CD4^+$T cells. (a) Correlations between double $CD107a^+$ interferon $(IFN)\gamma^+$ $CD8^+$T cells specific for the membrane (M) protein with viral load (left) and for the nucleocapsid (N) protein with interleukin (IL)-6 levels (right). Spearman rank correlation (n=33 for viral load and n=32 for IL-6). **(b)** Correlations between days to discharge since symptoms onset (top) or baseline IL-6 levels (bottom) and the frequency of $CD4^+$ (left) and $CD8^+$T cells (right) expressing IL-4 at baseline (unstimulated control). **(c)** Correlation between viral load and $CD4^+$T cells expressing IL-4 after PMA/Ionomycin stimulation. Two-sided spearman rank correlation (n=33 for viral load and n=46 for days to discharge since symptoms onset and IL-6). **(d)** Net frequency of IFNγ, CD107a, IL-4 and IL-10 expression in SARS-CoV-2-specific $CD4^+$T cells in response to all viral proteins (membrane (M), nucleocapsid (N) and spike (S)) based on $CXCR3^+CCR7^+$ and $CXCR3^+CCR7^-$ subsets for each individual patient (non-hospitalized n=14 in orange; mild n=20 in blue and severe n=12 in pink). Dots connected by the same line represent the same individual. Statistical comparisons were performed using two-sided non-parametric Wilcoxon matched-pairs signed rank test to compare the two groups ($CXCR3^+CCR7^+$ vs. $CXCR3^+CCR7^-$): IFNγ (p=0.022, p=0.0006 and p=0.0049), CD107a (p=0.042), IL-4 (p=0.019, p=0.0011 and p=0.042) and IL-10 (p=0.0001).

Supplementary Fig. 4

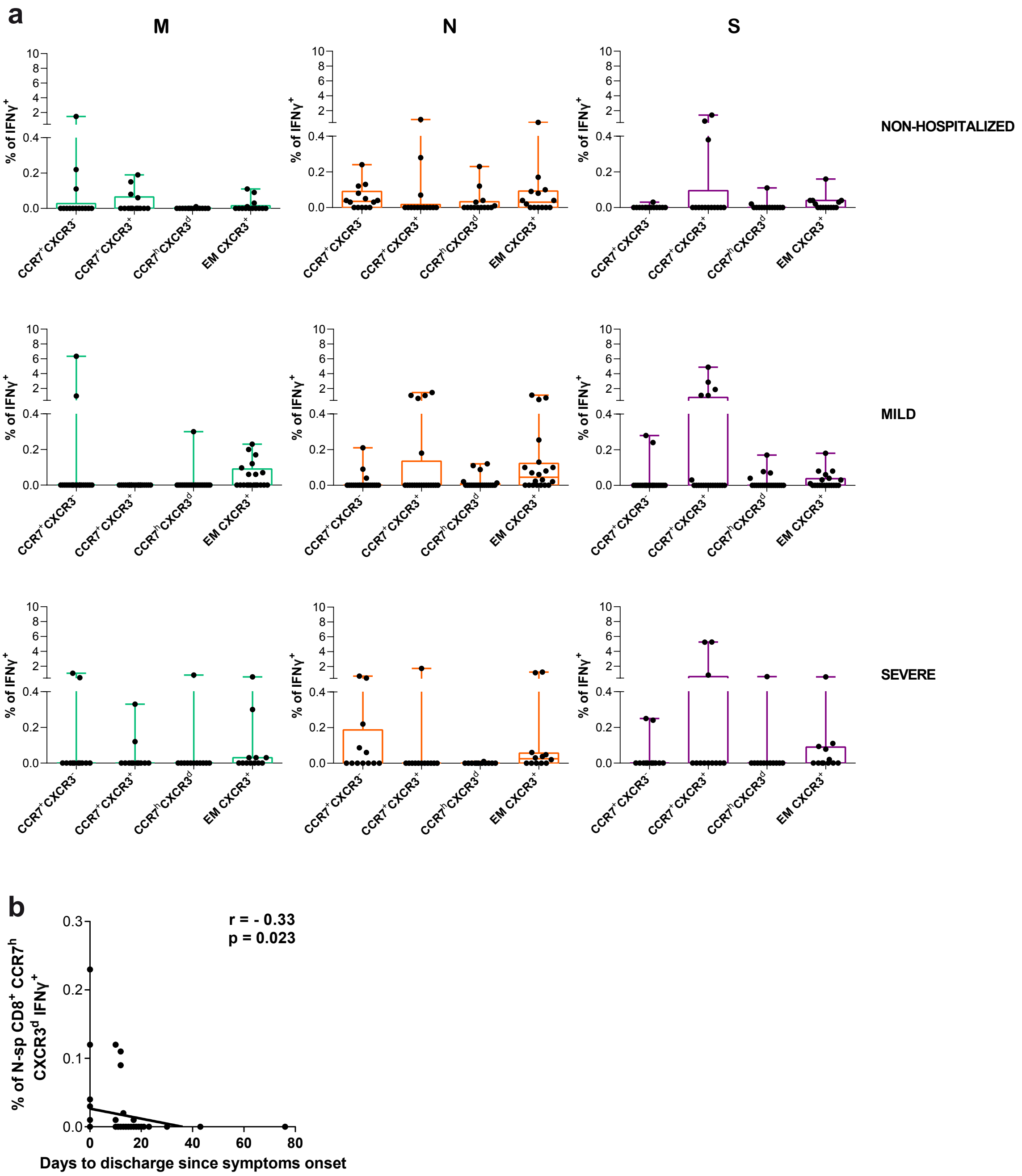

Supplementary Fig. 4. IFNγ expression in SARS-CoV-2-specific CD8$^+$ T cell subsets during acute infection. (a) Net frequency of interferon (IFN)γ expression in CCR7$^+$CXCR3$^-$, CCR7$^+$CXCR3$^+$, CCR7^{h}CXCR3^d and EM CXCR3$^+$ subsets within CD8$^+$ T cells after stimulation with any of the three viral proteins (membrane (M), nucleocapsid (N) and spike (S) proteins). Data are shown as median and upper range, where each dot represents an individual patient for each group (non-hospitalized n=14; mild n=20 and severe n=12). **(b)** Correlation between days to hospital discharge since symptoms onset and nucleocapsid-specific (N-sp) CD8$^+$ CCR7^{h}CXCR3^d IFNγ$^+$ T cells. Two-sided spearman rank correlation (n=46).

Supplementary Fig. 5

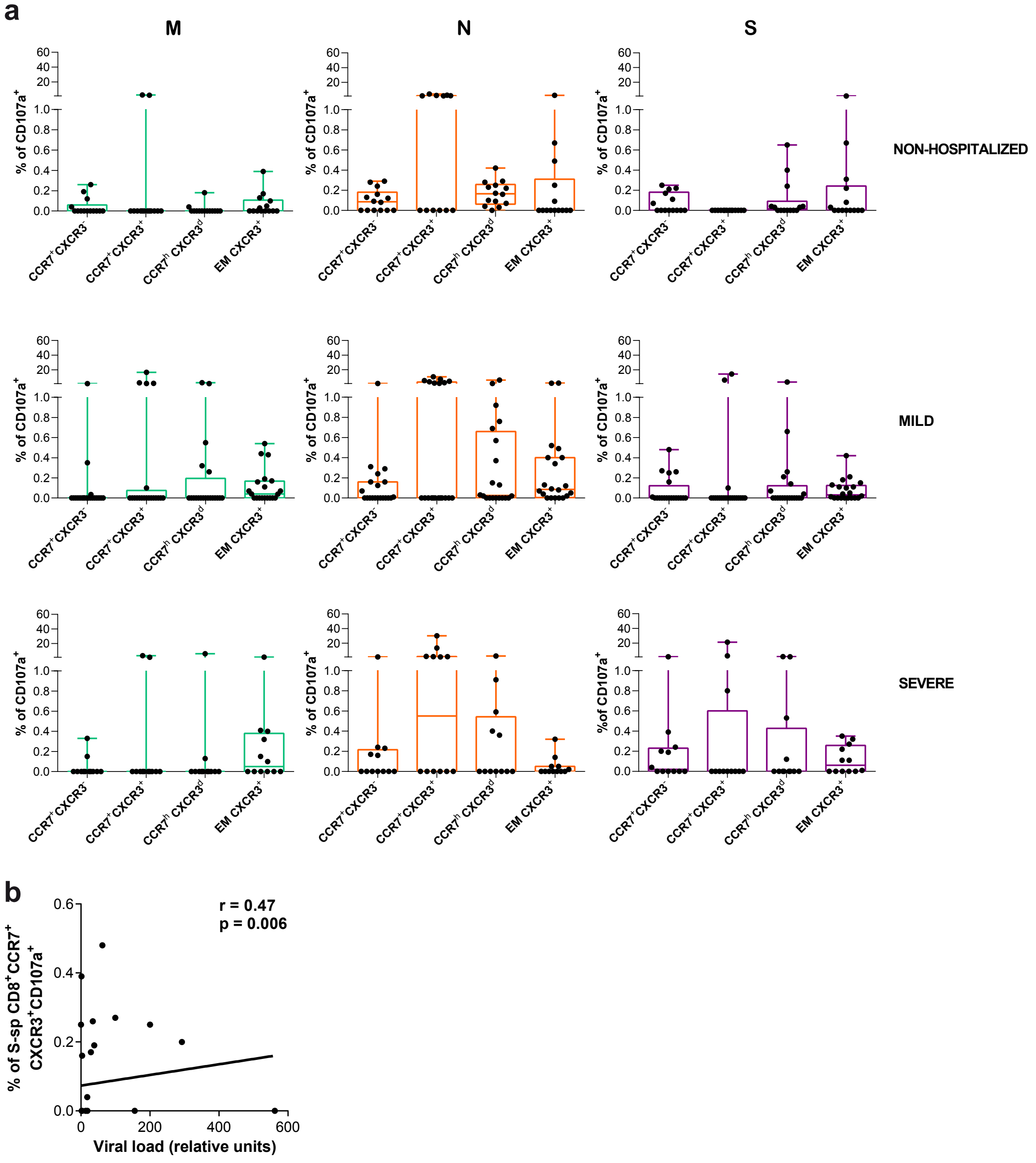

Supplementary Fig. 5. CD107a expression in SARS-CoV-2-specific CD8^{+} T cell subsets during acute infection. (a) Net frequency of CD107a expression in CCR7^{+}CXCR3^{-}, CCR7^{+}CXCR3^{+}, CCR7^{h}CXCR3^{d} and EM CXCR3^{+} subsets within CD8^{+} T cells after stimulation with any of the three viral proteins (membrane (M), nucleocapsid (N) and spike (S) proteins). Data are shown as median and upper range, where each dot represents an individual patient for each group (non-hospitalized n=14; mild n=20 and severe n=12). **(b)** Correlation between viral load and the frequency of spike-specific (S-sp) CD8^{+} CCR7^{+}CXCR3^{+} CD107a^{+} T cells. Two-sided spearman rank correlation (n=33).

Supplementary Fig. 6

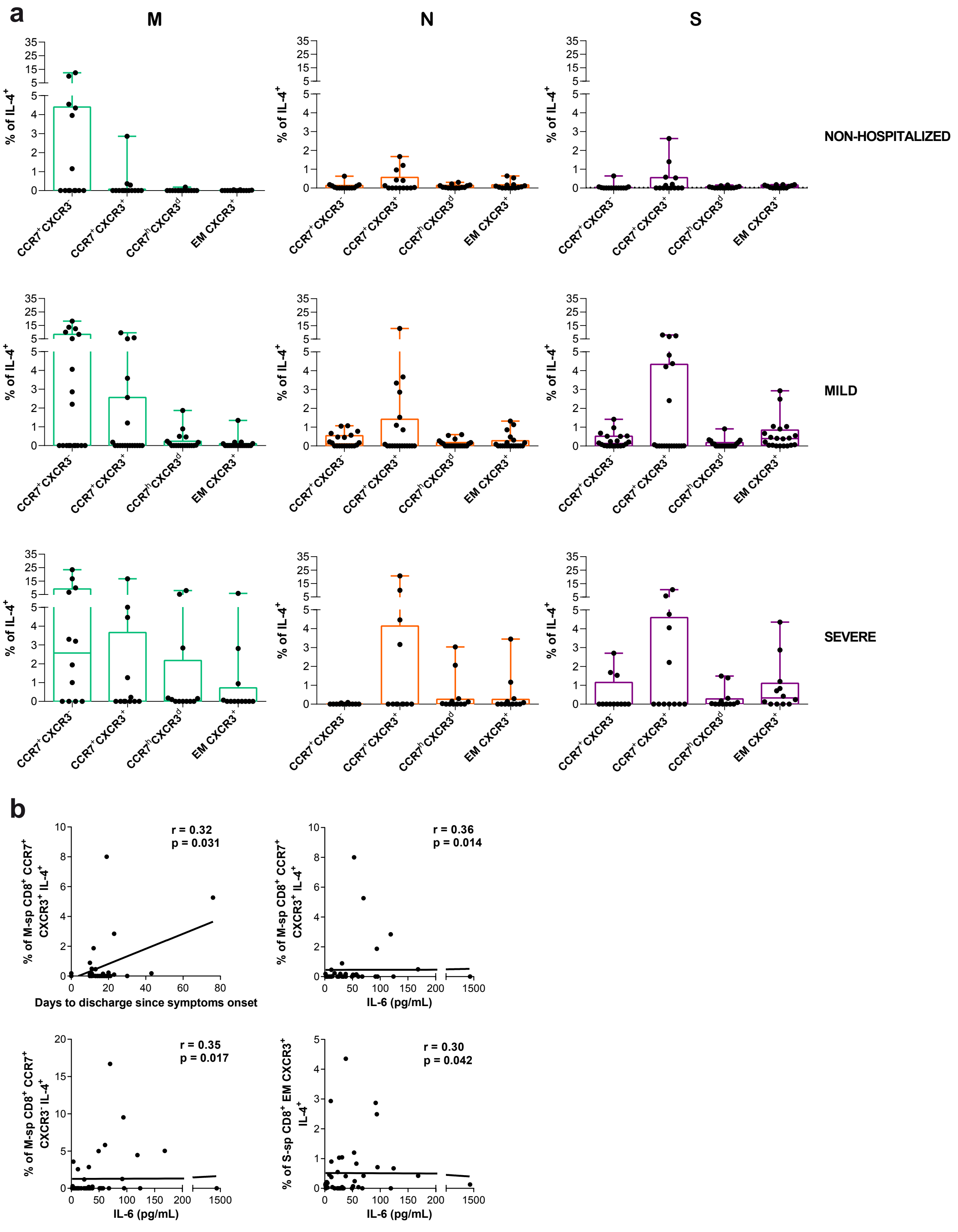

Supplementary Fig. 6. IL-4 expression in SARS-CoV-2-specific CD8$^+$ T cell subsets during acute infection. (a) Net frequency of interleukin (IL)-4 expression in CCR7$^+$CXCR3$^-$, CCR7$^+$CXCR3$^+$, CCR7^{h}CXCR3^d and EM CXCR3$^+$ subsets within CD8$^+$T cells after stimulation with any of the three viral proteins (membrane (M), nucleocapsid (N) and spike (S) proteins). Data are shown as median and upper range, where each dot represents an individual patient for each group (non-hospitalized n=14; mild n=20 and severe n=12). **(b)** Correlations between days to hospital discharge since symptoms onset or baseline IL-6 levels and the frequency of membrane (M-sp) or spike-specific (S-sp) CCR7/CXCR3 subsets of CD8$^+$ IL-4$^+$ T cells. Two-sided spearman rank correlation (n=46).

Supplementary Fig. 7

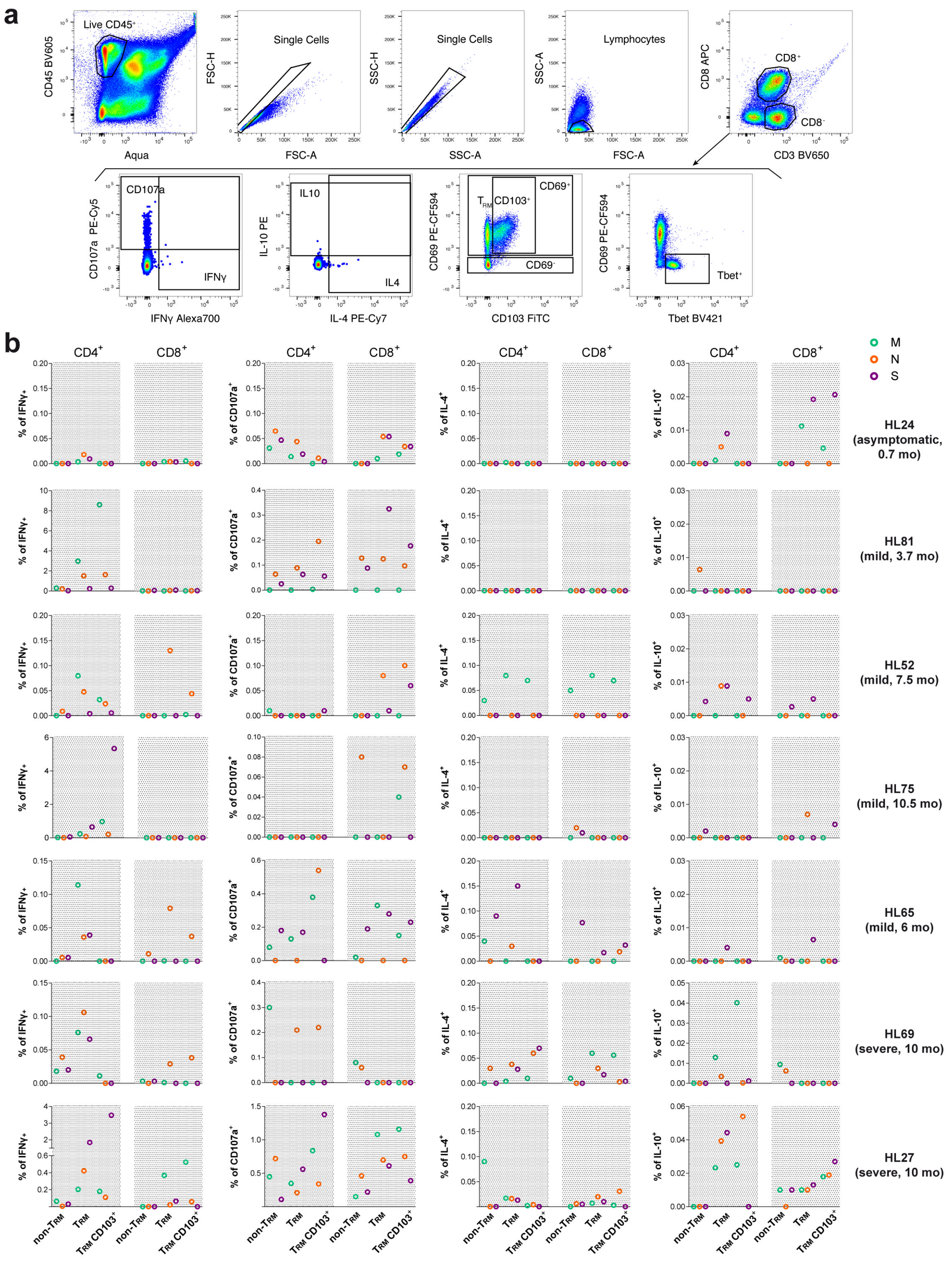

Supplementary Fig. 7. Functional analysis of lung-resident SARS-CoV-2-specific T cells. (a) Gating strategy employed for phenotypic analysis of lung-derived SARS-CoV-2-specific $CD4^+$ and $CD8^+$T cells presented on Figs. 6 and 7. Gating strategy consisted on a live $CD45^+$ gate, followed by doublet exclusion, a lymphocyte gate based on FSC vs. SSC and then a T cell gate based on $CD3^+$ $CD8^+$ or $CD8^-$ (putative $CD4^+$); from there we identified different functions (interferon (IFN)γ, CD107a, interleukin (IL)-4, IL-10) and resident phenotypes. Phenotype was based on the expression of CD69, CD103 and T-bet, where tissue-resident memory T cells (T_{RM}) were $CD69^+$, with a fraction of them expressing CD103, and non-T_{RM} were $CD69^-$. As shown, T-bet was associated to the non-T_{RM} fraction. **(b)** Net frequencies of SARS-CoV-2-specific $CD4^+$ or $CD8^+$ non-T_{RM}, T_{RM} and T_{RM} $CD103^+$ with different functions (IFNγ, CD107a, IL-4, IL-10) from patients HL24, HL81, HL52, HL75, HL65, HL69 and HL27. Patient cohort and months (mo) after first confirmatory SARS-CoV-2 RT-PCR are indicated. Viral proteins are shown in color green (membrane protein, M), orange (nucleocapsid protein, N) and purple (spike protein, S).

Supplementary Table 1. Patient characteristics at baseline.

	**Non-hospitalized** n=14	**Mild** n=20	**Severe** n=12	*P-value between groups*
Age (years), range [a]	25-42	38-64	51-69	***<0.0001***
Females, n (%) [b]	10 (71.4)	11 (55)	4 (33.3)	0.556
Fever, n (% yes) [b]	9 (64.2.3)	17 (85)	8 (66.6)	0.844
Dyspnea, n (% yes) [b]	2 (14.2)	15 (75)	7 (58.3)	0.101
SAFI ratio[c], median [interquartile range, IQR] [a]	475 [475-475]	466.0 [461-471]	447 [429.3-460]	***<0.0001***
SAFI ratio 48h, median [IQR] [a]	475 [475-475]	454.5 [448.3-461]	342.5 [283.5-396]	***<0.0001***
Days from symptoms to discharge, median [IQR] [a]	0 [0-0]	12.5 [11-15.7]	21 [17.5-28.2]	***<0.0001***
Charlson Comorbidity Index, median [IQR] [a]	0 [0-0]	1 [0-2]	2 [1.2-2]	***0.0002***
**Immunology and Virology**				
Leucocytes (10^9/L), median [IQR] [a]	6.1 [5.4-7.3]	5.7 [4.6-7.3]	7.2 [4.9-9.3]	0.462
Lymphocytes (%), median [IQR] [a]	33.5 [29.3-42.3]	21.7 [15-25.5]	15 [11.2-19.2]	***<0.0001***
Monocytes (%), median [IQR] [a]	7 [6.5-8.2]	8.2 [6.6-9.9]	5.7 [2.9-9.5]	0.243
Neutrophils (%), median [IQR] [a]	55.4 [49.2-61]	69.75 [61.88-77.9]	77.4 [64.6-79.75]	***0.0006***
Eosinophils (%), median [IQR] [a]	2.6 [1.3-5.1]	0.2 [0-0.7]	0 [0-0.07]	***<0.0001***
Platelets (10^9/L), median [IQR] [a]	291 [254.3-310.3]	185.5 [158.3-297]	191 [113.3-292.3]	0.073
Viral load (relative to housekeeping), median [IQR] [a]	0.2 [0.1-4.9]	3.5 [0.2-71.3]	3.32 [0.8-165.6]	0.215
**Biochemistry**				
D-dimer, (ng/ml), median [IQR] [a]	94 [54.7-122]	181 [130-401]	339 [247-965]	***0.0003***
Ferritin, (ng/ml), median [IQR] [a]	147 [30-202.0]	336 [164.8-537.8]	550.5 [309-1416]	***0.0002***
Interleukin (IL)-6, (pg/ml), median [IQR] [a]	1.7 [1.5-3]	25.6 [12.3-28.2]	55.3 [40.7-94.24]	***<0.0001***

Statistical comparisons between groups (non-hospitalized, mild and severe) were performed using [a]Kruskal-Wallis rank-sum test with Dunn's multiple comparison test (two-sided) and/or [b]Chi-square test. [c]SAFI ratio corresponds to the percentage of oxyhemoglobin saturation (SaO_2) in relation to the percentage of inspired oxygen (FiO_2) after 48h of hospitalization.

Supplementary Table 2. Spearman's correlation test between clinical parameters and the net frequency of SARS-CoV-2-specific CD4+ and CD8+T cells by function.

	Days to hospital discharge since symptoms onset (n=46)			Interleukin (IL)-6 (pg/ml) (n=46)			SAFI ratio[a] (48h) (n=46)		
**CD4+T cells**		*p-value*			*p-value*			*p-value*	
**M peptides[b]**	**Spear[c]. r**	**Non-adj.**	**FDR**	**Spear. r**	**Non-adj.**	**FDR**	**Spear. r**	**Non-adj.**	**FDR**
IFNγ[d]	0.39	***0.006***	0.050	0.45	***0.0013***	***0.013***	-0.55	***0.0001***	***0.0006***
CD107a	0.39	0.122	0.264	0.09	0.537	0.698	-0.32	***0.030***	0.091
IL-4[e]	0.05	0.711	0.816	-0.002	0.985	0.985	-0.005	0.973	0.982
IL-10	-0.32	***0.028***	0.135	-0.31	***0.036***	0.156	0.33	***0.022***	0.071
**N peptides**									
IFNγ	0.29	0.053	0.172	0.23	0.128	0.306	-0.42	***0.004***	***0.018***
CD107a	0.01	0.945	0.945	0.19	0.208	0.387	-0.25	0.088	0.172
IL-4	-0.07	0.624	0.761	-0.08	0.579	0.729	0.03	0.849	0.921
IL-10	-0.23	0.120	0.264	-0.22	0.144	0.311	0.33	***0.022***	0.072
**S peptides**									
IFNγ	0.39	***0.007***	0.055	0.35	***0.017***	0.094	-0.52	***0.0002***	***0.001***
CD107a	0.19	0.204	0.398	0.26	0.077	0.215	-0.43	***0.003***	***0.015***
IL-4	0.16	0.273	0.454	0.12	0.391	0.621	-0.19	0.203	0.375
IL-10	0.04	0.785	0.829	0.06	0.664	0.809	0.05	0.752	0.841
All **peptides**									
IFNγ	0.44	***0.002***	***0.026***	0.46	***0.001***	***0.013***	-0.62	***< 0.0001***	***0.0002***
CD107a	0.21	0.158	0.325	0.33	***0.024***	0.119	-0.52	***0.0002***	***0.001***
IL-4	0.12	0.417	0.623	0.11	0.441	0.662	-0.15	0.334	0.501
IL-10	-0.28	0.057	0.172	-0.22	0.133	0.306	0.35	***0.017***	0.067
**CD8+T cells**									
**M peptides**									
IFNγ	0.12	0.422	0.623	0.19	0.193	0.387	-0.12	0.413	0.591
CD107a	0.23	0.109	0.264	0.42	***0.003***	***0.030***	-0.24	0.113	0.209
IL-4	0.30	***0.037***	0.148	0.29	0.050	0.163	-0.29	***0.048***	0.135
IL-10	-0.07	0.623	0.761	0.12	0.424	0.661	0.08	0.582	0.732
**N peptides**									
IFNγ	0.15	0.324	0.574	-0.19	0.204	0.387	0.02	0.877	0.925
CD107a	-0.17	0.254	0.473	-0.18	0.226	0.402	0.26	0.081	0.172
IL-4	0.04	0.766	0.829	-0.04	0.787	0.903	-0.09	0.524	0.704
IL-10	-0.11	0.431	0.622	-0.16	0.273	0.454	0.12	0.424	0.591
S **peptides**									
IFNγ	0.31	***0.038***	0.148	0.29	***0.044***	0.158	-0.26	0.080	0.172
CD107a	0.12	0.393	0.623	0.01	0.927	0.985	0.09	0.550	0.716
IL-4	0.48	***0.0006***	***0.010***	0.51	***0.0002***	***0.008***	-0.58	***< 0.0001***	***0.0003***
IL-10	0.09	0.552	0.743	0.12	0.407	0.661	-0.07	0.657	0.776
All **peptides**									
IFNγ	0.35	***0.015***	0.103	0.09	0.537	0.698	-0.26	0.078	0.172
CD107a	-0.03	0.816	0.838	0.01	0.946	0.985	0.16	0.296	0.481
IL-4	0.27	0.061	0.172	0.29	***0.042***	0.158	-0.35	***0.016***	0.067
IL-10	-0.12	0.394	0.622	0.004	0.974	0.985	0.15	0.332	0.501

[a]SAFI ratio corresponds to the percentage of oxyhemoglobin saturation (SaO_2) in relation to the percentage of inspired oxygen (FiO_2) after 48h of hospitalization. [b]Viral peptides (membrane (M), nucleocapsid (N) and spike (S)); [c]Spear (two-sided spearman rank correlation): non-adj (non-adjusted for multiple comparisons) or FDR (false discovery rate, adjusted for multiple comparisons); [d]Interferon (IFN); [e]Interleukin (IL)

Supplementary Table 3. Characteristics of convalescent COVID-19 patients from lung resections.

ID	Age (range)	Sex	Baseline Interleukin-6 (pg/ml)	Days of hospitalization	Months since positive RT-PCR	Group
HL24	20-40	Male	-	0	0.7	Asymptomatic
HL27	60-80	Male	133.7	30	10	Severe-PCR+ for 2 months
HL52	60-80	Male	26.15	5	7.5	Mild
HL65	40-60	Female	5.25	3	6	Mild-PCR+ for 4 months
HL69	60-80	Male	34.66	35	10	Severe
HL75	60-80	Male	9.13	13	10.5	Mild
HL81	60-80	Male	309	9	3.7	Mild

Supplementary Table 4. Real Time RT-PCR Primers and Probes.

Name	Description	Oligonucleotide Sequence (5’>3’)
2019-nCoV_N1-F	2019-nCoV_N1 Forward Primer	GAC CCC AAA ATC AGC GAA AT
2019-nCoV_N1-R	2019-nCoV_N1 Reverse Primer	TCT GGT TAC TGC CAG TTG AAT CTG
2019-nCoV_N1-P	2019-nCoV_N1 Probe	FAM-ACC CCG CAT TAC GTT TGG TGG ACC-BHQ1
RP-F	RNAse P Forward Primer	AGA TTT GGA CCT GCG AGC G
RP-R	RNAse P Reverse Primer	GAG CGG CTG TCT CCA CAA GT
RP-P	RNAse P Probe	FAM-TTC TGA CCT GAA GGC TCT GCG CG-BHQ-1
