# Supplementary material for: Peripheral and lung resident memory T cell responses against SARS-CoV-2
Source: Nat Commun. 2021 May 21;12:3010. doi: 10.1038/s41467-021-23333-3 (PMC8140108; doi:10.1038/s41467-021-23333-3)
Supplement: Supplementary file 3 — Reporting Summary [file 41467_2021_23333_MOESM3_ESM.pdf]

nature research

Corresponding author(s): Maria José Buzón, Meritxell Genescà Ferrer

Last updated by author(s): Apr 12, 2021

Reporting Summary

Nature Research wishes to improve the reproducibility of the work that we publish. This form provides structure for consistency and transparency in reporting. For further information on Nature Research policies, see our Editorial Policies and the Editorial Policy Checklist.

Statistics

For all statistical analyses, confirm that the following items are present in the figure legend, table legend, main text, or Methods section.

n/a	Confirmed	
☐	☒	The exact sample size (n) for each experimental group/condition, given as a discrete number and unit of measurement
☐	☒	A statement on whether measurements were taken from distinct samples or whether the same sample was measured repeatedly
☐	☒	The statistical test(s) used AND whether they are one- or two-sided<br>*Only common tests should be described solely by name; describe more complex techniques in the Methods section.*
☐	☒	A description of all covariates tested
☐	☒	A description of any assumptions or corrections, such as tests of normality and adjustment for multiple comparisons
☐	☒	A full description of the statistical parameters including central tendency (e.g. means) or other basic estimates (e.g. regression coefficient) AND variation (e.g. standard deviation) or associated estimates of uncertainty (e.g. confidence intervals)
☒	☐	For null hypothesis testing, the test statistic (e.g. F, t, r) with confidence intervals, effect sizes, degrees of freedom and P value noted<br>*Give P values as exact values whenever suitable.*
☒	☐	For Bayesian analysis, information on the choice of priors and Markov chain Monte Carlo settings
☒	☐	For hierarchical and complex designs, identification of the appropriate level for tests and full reporting of outcomes
☒	☐	Estimates of effect sizes (e.g. Cohen's d, Pearson's r), indicating how they were calculated

Our web collection on statistics for biologists contains articles on many of the points above.

Software and code

Policy information about availability of computer code

Data collection	FACS Diva software 6.2. (firmware version 1.2) was used for flow cytometry sample acquisition.
Data analysis	We used GraphPad Prism (version 7.0) for statistical analysis and FlowJo vX.0.7 (TreeStar) software for flow cytometry.

For manuscripts utilizing custom algorithms or software that are central to the research but not yet described in published literature, software must be made available to editors and reviewers. We strongly encourage code deposition in a community repository (e.g. GitHub). See the Nature Research guidelines for submitting code & software for further information.

Data

Policy information about availability of data

All manuscripts must include a data availability statement. This statement should provide the following information, where applicable:

- Accession codes, unique identifiers, or web links for publicly available datasets
- A list of figures that have associated raw data
- A description of any restrictions on data availability

The authors declare that the data supporting the findings of this study are available within the paper and its supplementary information files. There are no restriction for any materials used in this study.

Field-specific reporting

Life sciences study design

All studies must disclose on these points even when the disclosure is negative.

Sample size	No sample-size calculations were performed. Based on tissue availability, sample size was determined to be adequate based on the magnitude and consistency of measurable differences between groups.
Data exclusions	Samples from adult patients diagnosed with acute COVID-19 defined by symptomatology and/or clinical findings and confirmed by positive reverse-transcriptase polymerase chain reaction (RT-PCR) for SARS-CoV-2, obtained between 7 and 16 days after symptoms onset, were included in the study. No data exclusion were applied.
Replication	Replication was not applied to the study due to sample limitation.
Randomization	Normal donors were included as controls to each experimental batch analyses. According to disease severity patients, at the discretion of the treating physician, patients were classified in three groups:<br>a) Patients with severe disease: individuals with radiologically confirmed pneumonia that required hospitalization and had acute respiratory failure and/or analytical parameters of severity and/or extensive radiological involvement.<br>b) Patients with mild disease: individuals with radiologically confirmed pneumonia that required hospital admission but without criteria of severity.<br>c) Non-hospitalized patients: individuals without pneumonia and with pauci-asymptomatic disease that did not require hospitalization and managed on an outpatient clinic.
Blinding	Investigators were blinded to group allocation both during data collection as well for studies involving lung and blood samples.

Reporting for specific materials, systems and methods

We require information from authors about some types of materials, experimental systems and methods used in many studies. Here, indicate whether each material, system or method listed is relevant to your study. If you are not sure if a list item applies to your research, read the appropriate section before selecting a response.

Materials & experimental systems

n/a	Involved in the study	
☐	☒	Antibodies
☒	☐	Eukaryotic cell lines
☒	☐	Palaeontology and archaeology
☒	☐	Animals and other organisms
☐	☒	Human research participants
☒	☐	Clinical data
☒	☐	Dual use research of concern

Methods

n/a	Involved in the study	
☒	☐	ChIP-seq
☐	☒	Flow cytometry
☒	☐	MRI-based neuroimaging

Antibodies

Antibodies used	Anti-CD107a (PE-Cy5, BD Biosciences, Cat# 555802, clone H4A3), anti-CD3 (Per-CP, BD Biosciences, Cat# 340663, clone SK7), anti-CD4 (BV605, BD Biosciences, Cat# 562658, clone RPA-T4), anti-CD56 (FITC, BD Biosciences, Cat# 562794, clone B159), anti-Caspase-3 (AF647, BD Biosciences, Cat# 560626, clone C92-605), anti-Bcl-2 (BV421, Biolegend, Cat# 658709, clone 100), anti-IL-4 (PE-Cy7, eBioscience, Cat# 25-7049-82, clone 8D4-8), anti-IL-10 (PE, BD Biosciences, Cat# 559330, clone JES3-19F1), anti-IFNg (AF700, Invitrogen, Cat# MHCIFG29, clone B27), anti-CD103 (FITC, Biolegend, Cat# 350204, clone Ber-ACT8), anti-CD69 (PE-CF594, BD Biosciences, Cat# 562617, clone FN50), anti-CD40 (APC-Cy7, Biolegend, Cat# 313017, clone HB14), anti-CD8 (APC, BD Biosciences, Cat# 561952, clone RPA-T8), anti-CD3 (BV650, BD Biosciences, Cat# 563851, clone UCHT1), anti-CD45 (BV605, BD Biosciences, Cat# 564047, clone HI30), anti-T-bet (BV421, Biolegend, Cat# 644815, clone 4B10), anti-CD197 (CCR7, PE-CF594, BD Biosciences, Cat#562381, clone 150503) and anti-CD186 (CXCR3, Biolegend, Cat#353730, clone G025H7).
Validation	All antibodies have been tested for reactivity against the appropiate species on the specification sheets on the providers' websites or in published articles.<br>According to the manufacturer's website, anti-CD107a (clone H4A3) antibody (BD Biosciences, Cat#555802) has been routinely tested on the fixed and permeabilized Jurkat cells by flow cytometry with Cytofix/Cytoperm (Cat. No. 554714) for fixation and permeabilization. This antibody was previously validated in Chen JW et al., 1988, J Biol Chem.<br>According to the manufacturer's website, anti-CD3 (clone SK7) antibody (BD Biosciences, Cat# 562658) is intended for in vitro diagnostic use in the identification of cells expressing the CD3 antigen, using a BD FACS™ brand flow cytometer. This antibody was previously validated in van Dongen JJM et al., 1988, Blood.<br>According to the manufacturer's website, anti-CD4 (clone RPA-T4) antibody (BD Biosciences, Cat# 562658) is suitable for flow cytometry. Flow cytometric analysis of CD4 expression on human peripheral blood lymphocytes is provided on the website.<br>According to the manufacturer's website, anti-CD56 (clone B159) antibody (BD Biosciences, Cat# 562794) is suitable for flow cytometry. Flow cytometric analysis of CD56 expression on human peripheral lymphocytes is provided on the website.<br>According to the manufacturer's website, anti-Caspase-3 (clone C92-605) antibody (BD Biosciences, Cat# 560626) is suitable for

intracellular staining in flow cytometry. Flow cytometric analysis of apoptotic and non-apoptotic populations for active caspase-3 in Jurkat cells is provided on the website.
According to the manufacturer's website, anti-Bcl-2 (clone 100) antibody (Biolegend, Cat# 658709) is suitable for intracellular immunofluorescent staining with flow cytometric analysis. Flow cytometric analysis of Bcl-2 expression on human peripheral blood lymphocytes is provided on the website.
According to the manufacturer's website, anti-IL-4 (clone 8D4-8) antibody (eBioscience, Cat# 25-7049-82) is suitable for flow cytometry. Flow cytometric analysis of IL-4 expression on human peripheral cells is provided on the website.
According to the manufacturer's website, anti-IL-10 (clone JES3-19F1) antibody (BD Biosciences, Cat# 559330) has been routinely tested on fixed and permeabilized human peripheral blood lymphocytes by flow cytometry. Flow cytometric analysis of IL-10 expression by stimulated CD14+ human monocytes is provided on the website.
According to the manufacturer's website, anti-IFNg (clone B27) antibody (Invitrogen, Cat# MHCIFG29) is suitable for flow cytometry. This antibody was previously validated by Demers KR et al., 2016, PLos pathogens.
According to the manufacturer's website, anti-CD103 (clone Ber-ACT8) antibody (Biolegend, Cat# 350204) is quality control tested by immunofluorescent staining with flow cytometric analysis. Flow cytometric analysis of CD103 expression in human peripheral blood mononuclear cells is provided on the website.
According to the manufacturer's website, anti-CD69 (clone FN50) antibody (BD Biosciences, Cat#562617) is suitable for flow cytometry. Flow cytometric analysis of CD69 expression by stimulated peripheral blood mononuclear cells is provided on the website.
According to the manufacturer's website, anti-CD40 (clone HB14) antibody (Biolegend, Cat#313017) is quality control tested by immunofluorescent staining with flow cytometric analysis. Flow cytometric analysis of CD40 expression in human peripheral blood lymphocytes is provided on the website.
According to the manufacturer's website, anti-CD8 (clone RPA-T8) antibody (BD Biosciences, Cat#561952) is routinely tested for flow cytometry. Flow cytometric analysis of CD8 expression on human peripheral blood lymphocytes is provided on the website.
According to the manufacturer's website, anti-CD3 (clone UCHT1) antibody (BD Biosciences, Cat#563851) is routinely tested for flow cytometry. Flow cytometric analysis of CD3 expression on human peripheral blood lymphocytes is provided on the website.
According to the manufacturer's website, anti-CD45 (clone HI30) antibody (BD Biosciences, Cat#564047) is routinely tested for flow cytometry. Flow cytometric analysis of CD45 expression on human peripheral blood lymphocytes is provided on the website.
According to the manufacturer's website, anti-T-bet (clone 4B10) antibody (Biolegend, Cat# 644815) is quality tested for intracellular immunofluorescent staining with flow cytometric analysis. Flow cytometric analysis of T-bet expression on human peripheral blood lymphocytes is provided on the website.
According to the manufacturer's website, anti-CD197 (CCR7, clone 150503) antibody (BD Biosciences, Cat# 562381) is routinely tested for flow cytometry. Flow cytometric analysis of CD197 (CCR7) expression on human peripheral blood lymphocytes is provided on the website.
According to the manufacturer's website, anti-CD186 (CXCR3, clone G025H7) antibody (Biolegend, Cat# 353730) is quality tested by immunofluorescent staining with flow cytometric analysis. This antibody was previously validated in Kenderes KJ, et al., 2018, Cell Rep.

Human research participants

Policy information about studies involving human research participants

Population characteristics

Adult patients, 18 years old and older, diagnosed with acute COVID-19 were recruited at the Vall d'Hebron Hospital during the first COVID-19 outbreak between March and May 2020. Diagnosis of acute COVID-19 was defined by symptomatology and/or clinical findings and confirmed by positive reverse-transcriptase polymerase chain reaction (RT-PCR) for SARS-CoV-2 in a respiratory tract specimen. The study cohort consisted of patients with severe disease, with mild disease and non-hospitalized individuals. Patient information is summarized in Table S1. According to disease severity patients, at the discretion of the treating physician, patients were classified in three groups: (a)Patients with severe disease, (b)Patients with mild disease and (c) non-hospitalized patients. Blood samples from healthy adult donors were obtained via phlebotomy and were used as controls.
Lung biopsies were obtained from 5 patients recovered from SARS-CoV-2 infection who needed a lung resection. Samples from two patients with mild disease, two with severe disease and one asymptomatic patient were included in the study. All patientes tested negative for RT-PCR for SARS-CoV-2 before thoracic surgery. Patient information is summarized in materials and methods.

Recruitment

Patients who matched the above characteristics were approached and recruited into the studies without any additional bias. Blood samples from patients diagnosed with COVID-19 were recuited at the Vall d'Hebron Hospital by collaborating with the Infectious Disease Unit (from HUVH). 20 milliliters of blood were collected at baseline by phlebotomy in two EDTA tubes and stored at room temperature briefly prior to processing for PBMC and plasma isolation. Immunocompromised patients in which the immune response may be affected were excluded of the study. Samples were obtained between 7 and 16 days after symptoms onset.
Blood samples from healthy adult donors were also obtained via phlebotomy. These blood samples were collected for studies unrelated to COVID-19 between September 2018 and June 2019 (PR(AG)116/2018 and PR(AG)117-2018). At the time of enrollment in the initial studies, all individual donors provided informed consent that their samples could be used for future studies. These samples were considered to be from unexposed controls given that SARS-CoV-2 emerged as a novel pathogen in December 2019 and these samples were largely collected before this date. These donors were considered healthy in that they had no known history of any significant systemic illnesses. The cohort of healthy donors includes 12 individuals.
Lung samples from recovered SARS-CoV-2 donors were recruited based on surgical planification. Patients undergoing lung resections were recruited by collaborating thoracic surgeons at Hospital Universitari Vall d'Hebron (HUVH) in Barcelona, Spain. Concomitant to the lung biopsy, blood samples were also collected.

Ethics oversight

Study protocols were performed in accordance with the Declaration of Helsinki and approved by the corresponding Institutional Review Board (PR(AG)192/2020, PR(AG)212/2020, PR(AG)116/2018 and PR(AG)117-2018) of the Vall d'Hebron

University Hospital (HUVH), Barcelona, Spain. Written informed consent was provided by all patients recruited to this study and samples were prospectively collected and cryopreserved in the Vall d'Hebron Research Institute.

Note that full information on the approval of the study protocol must also be provided in the manuscript.

Flow Cytometry

Plots

Confirm that:

- [x] The axis labels state the marker and fluorochrome used (e.g. CD4-FITC).
- [x] The axis scales are clearly visible. Include numbers along axes only for bottom left plot of group (a 'group' is an analysis of identical markers).
- [x] All plots are contour plots with outliers or pseudocolor plots.
- [x] A numerical value for number of cells or percentage (with statistics) is provided.

Methodology

Sample preparation	PBMC were isolated by Ficoll density gradient centrifugation and stained as indicated in materials and methods section. Lung tissue was digested with an appropiated protocol (indicated in materials and methods section), stained with viability dye, then stained with indicated antibodies and resuspended in 1% paraformaldehyde for fix them before acquisition on an analyzer.
Instrument	BD FACS Fortessa analyzer.
Software	FACS Diva for data collection and FlowJo software for data analysis.
Cell population abundance	No sorting experiments were performed.
Gating strategy	All samples were initially gated using forward scatter and side scatter to identify events corresponding to cells (from blood samples) or, alternatively by CD45 and viability dye to identify events corresponding to lived hemapoietic cells (from lung biopsies), and then using forward scatter height vs. area and side scatter height vs. area to enrich for single cells, next alive cells were selected by negativity for viability dye (for blood samples). The follow gating steps are presented in principal and supplementary figures.

- [x] Tick this box to confirm that a figure exemplifying the gating strategy is provided in the Supplementary Information.
